# Synbiotic Microencapsulation of *Lactobacillus* Strains from Mexican Fermented Beverages for Enhanced Probiotic Functionality

**DOI:** 10.3390/molecules30051185

**Published:** 2025-03-06

**Authors:** Morayma Ramírez-Damián, Cynthia Garfias-Noguez, Luis G. Bermúdez-Humarán, María Elena Sánchez-Pardo

**Affiliations:** 1 Instituto Politécnico Nacional, Escuela Nacional de Ciencias Biológicas, Departamento de Ingeniería Bioquímica, Unidad Profesional Adolfo López Mateos, Zacatenco, Av. Wilfrido Massieu 399, Col. Nueva Industrial Vallejo, Alcaldía Gustavo A. Madero, Ciudad de México C.P. 07738, Mexico; mramirezd1601@alumno.ipn.mx (M.R.-D.); cgarfez@gmail.com (C.G.-N.); 2 INRAE, AgroParisTech, Micalis Institute, Université Paris-Saclay, Domain de Vilvert, 78350 Jouy-en-Josas, France; luis.bermudez@inrae.fr

**Keywords:** synbiotic, spray-drying, probiotic functionality, antioxidant activity

## Abstract

Synbiotics, which combine probiotics and prebiotics, represent an innovative approach to developing functional foods with enhanced health benefits compared to their individual components. This study focuses on the production of synbiotics through the microencapsulation of *Lactobacillus* strains isolated from traditional Mexican fermented beverages, contributing to the advancement of technologies for functional food development. Three *Lactobacillus* strains (*Lacticaseibacillus rhamnosus* LM07, *Lactiplantibacillus plantarum* LM19, and *Levilactobacillus brevis* LBH1070) were microencapsulated by spray-drying using a mixture of maltodextrin and gum arabic as wall materials and inulin as a prebiotic. The microencapsulation process achieved high survival rates (>90%), low moisture content (~5%), and low water activity (~0.3), ensuring long-term stability. Notably, the microencapsulated strains demonstrated improved tolerance to gastrointestinal conditions, enhanced adhesion properties, and increased antioxidant activity compared to non-microencapsulated strains. These results highlight the potential of microencapsulation as an innovative technology not only to preserve but also to enhance probiotic properties, facilitating the development of functional foods with improved health-promoting properties, extended shelf life, and stability at room temperature.

## 1. Introduction

During the past decades, there has been an increased interest in developing products that are attractive in terms of taste to the consumer but can also offer added value with extra benefits for their health, such as functional foods. To develop these products, research into different foods has been expanded, including fermented foods, which are produced through biochemical changes through the controlled growth of microorganisms [1]. In Mexico, there is an extensive list of different traditional fermented beverages produced throughout the country, such as tequila, mezcal, pulque, tejuino, pozol, and tepache. It has been reported that the consumption of various traditional Mexican beverages has beneficial effects on health, which have been attributed to the fermentation process where diverse microorganisms play an important role. Therefore, the isolation and characterization of microorganisms involved in the production processes of these beverages, especially those with probiotic potential, have aroused the interest of researchers [1].

According to the FAO, probiotics are microorganisms capable of conferring benefits on the host if administered in sufficient quantity. On the other hand, prebiotics are indigestible components of food that help improve the host’s health conditions by stimulating the growth of microorganisms that inhabit the host’s gastrointestinal region [2]. Therefore, synbiotics, a combination of both components (probiotic and prebiotic), should confer more health benefits than their individual use [3]. These benefits are specific to each strain, and for them to be reflected in the host’s health, it is necessary to consider the survival and dosage of bacteria in the food matrix. Generally, it is recommended to maintain a survival of ~10^7^ colony-forming units (CFUs)/mL for a commercial product shelf life [4].

One of the main challenges in probiotic applications is ensuring their stability under harsh conditions, such as in stomach acid and bile salts, while also maintaining their ability to adhere to the intestinal mucosa, colonize the gastrointestinal tract, produce antimicrobial compounds, and sustain metabolic activity in the intestine. Their incorporation into food products is particularly challenging due to factors that can compromise survival and viability during production, storage, distribution, and consumption, including oxygen exposure, pH fluctuations, and temperature variations [5]. To address these challenges, various protective mechanisms have been explored to enhance probiotic resistance to adverse conditions. These approaches aim to reduce cell loss, maintain viability, and enable a gradual release of probiotics, thereby facilitating colonization and metabolic activity in the intestinal tract for a significant health impact [5,6,7].

Encapsulation techniques have been widely used to protect probiotics and extend their stability during storage. Among these, spray-drying microencapsulation is a commonly employed method in the food industry due to its reproducibility, its high encapsulation efficiency, and the enhanced stability of the final product [2].

The choice of encapsulating agent is crucial for maintaining microcapsule stability. The selected material must be non-toxic, protect encapsulated cells from harmful environmental factors, optimize the absorption of probiotic compounds, and control their release during gastrointestinal transit. Additionally, it should ensure high core material retention within the powder particles, enhance microcapsule stability, and extend product shelf life [2,5,8].

Various food-grade polymers—including carbohydrates, proteins, and lipids—have been used in probiotic encapsulation due to their non-toxicity, high compatibility, and ability to form protective gels. Carbohydrate-based polymers with high activation energy are particularly effective in preventing heat and oxidative stress during storage [5]. Maltodextrin (MD), for example, is a polymer derived from the partial hydrolysis of starch. It consists of D-glucose units primarily linked by α-(1→4) glycosidic bonds, with some branched segments linked by α-(1→6) glycosidic bonds [9]. It is widely used in microencapsulation due to its non-toxicity, low cost, excellent solubility, and low viscosity, even at high solid concentrations. Additionally, maltodextrin is readily available and provides moderate prebiotic benefits. However, due to its low emulsifying capacity, it is often combined with other encapsulating agents, such as gum arabic. Gum arabic (GA) is a complex of sugars and hemicelluloses derived from *Acacia senegal* (L.), consisting of an arabinonic acid core bound to calcium, magnesium, and potassium, along with D-galactose, L-arabinose, and L-rhamnose [9]. At low concentrations (1–10%), GA acts as a film-forming agent, reduces hygroscopicity and powder caking, prevents the complete dehydration of cellular components, and stabilizes bacterial cells during drying and storage [10]. Moreover, GA has been reported to exhibit prebiotic activity by selectively stimulating beneficial gut bacteria such as *Bifidobacterium* and *Lactobacillus*, contributing to intestinal health and metabolic regulation [11]. Additionally, inulin has been widely used for probiotic microencapsulation in spray-drying processes. Inulin is composed of D-fructose units linked by (2→1)-β-glycosidic bonds, with a terminal glucose molecule. In the food industry, inulin is commonly used as a sweetener, stabilizer, and prebiotic. Studies have shown that inulin provides protection against inflammatory and colonic diseases in animals. Moreover, its consumption has been associated with reduced triacylglycerol levels, lower risk of atherosclerosis, decreased incidence of intestinal disorders (e.g., irritable bowel syndrome and colon cancer), and improved calcium, magnesium, and iron absorption [9,12,13,14]

Several studies have evaluated the microencapsulation of different strains of lactobacilli and their viability during digestion, as well as their stability during storage. Russo et al. [15] encapsulated *Lacticaseibacillus fermentum* CRL1446, *Lacticaseibacillus johnsonii* CRL1231, and *Lactobacillus acidophilus* CRL1014 in sodium alginate, inulin, and maltodextrin. They evaluated the viability of the strains during the digestion process and their stability when refrigerated for up to 12 months. Xavier dos Santos et al. [16] studied *Lactobacillus acidophilus* La-5 encapsulated in inulin, fructooligosaccharides, and skim milk, analyzing its resistance to digestive conditions, although no data were reported regarding stability during storage. Zhu et al. [17] microencapsulated *Lactiplantibacillus plantarum* BM-1 in reconstituted skim milk and sucrose. In this study, a significant decrease in viability was observed after digestion, and the stability of the strain was evaluated for only two months of storage. Páez et al. [18] conducted the microencapsulation of *Lacticaseibacillus casei* Nad, *Lacticaseibacillus paracasei* A13, *Lactobacillus acidophilus* A9, *Lactiplantibacillus plantarum* com, and *Lactiplantibacillus plantarum* 8329 in different formulations with skim milk, starch, and whey protein concentrate. The results indicated a higher survival rate under digestive conditions, with storage reported for up to 75 days at 5 °C, 25 °C, and 37 °C. Rosolen et al. [19] microencapsulated *Lactococcus lactis* subsp. *lactis* R7 in whey, inulin, and aerosil. It was reported that the viability of the strain was not affected by digestive conditions, and its stability was evaluated over six months at −20 °C, 4 °C, and 25 °C. Avila Reyes et al. [20] investigated the microencapsulation of *Lacticaseibacillus rhamnosus* B442 in starch and inulin, determining its stability for 32 days at 4 °C and 25 °C. Finally, Arepally et al. [10] reported the microencapsulation of *Lactobacillus acidophilus* NCDC 016 in maltodextrin and different concentrations of gum arabic. Protection of the strain under gastrointestinal conditions was observed, and its stability was reported for three months at 4 °C and 25 °C.

While the studies mentioned above demonstrate the potential of microencapsulation for preserving the viability of lactobacilli strains, limited research has focused on strains isolated from traditional fermented beverages. Furthermore, while previous studies have shown the protective effects of encapsulation, few have evaluated synbiotic formulations capable of maintaining viability at 10^7^ CFU over extended periods without refrigeration. In addition, the effects of microencapsulation on key probiotic properties, such as adhesion and antioxidant activity, remain largely unexplored.

To address these gaps, this study aimed to develop and evaluate three synbiotic formulations of Lactobacillus through spray-drying microencapsulation, using maltodextrin and gum arabic as wall materials and inulin as a prebiotic. The goal was to enhance the stability and viability of the encapsulated strains and assess the effects of microencapsulation on their key functional properties. The Lactobacillus strains used in this study were previously isolated from the fermentation of traditional Mexican beverages. Specifically, *Lacticaseibacillus rhamnosus* LM07 and *Lactiplantibacillus plantarum* LM19 were obtained from the agave fermentation stage in mezcal production in Oaxaca, Mexico, as reported by Hernández-Delgado et al. [21], while *Levilactobacillus brevis* LBH1070 was isolated from pulque (*xaxtle*) in Tlaxcala, Mexico, by Torres-Maravilla et al. [22]. The proportions of maltodextrin, gum arabic, and inulin in the formulation were determined through prior unpublished experimentation, detailed in European Patent Application No. EP24306864.0. The findings of this study contribute to the growing body of knowledge on probiotic stabilization and may provide valuable insights for the development of novel functional food products.

## 2. Results

### 2.1. Microencapsulation

The drying conditions used in this study resulted in microencapsulation process yields of 72.2 ± 4.9% for *L. rhamnosus* LM07, 69.0 ± 2.5% for *L. plantarum* LM19, and 71.7 ± 4.1% for *L. brevis* LBH1070. The synbiotics obtained after microencapsulation exhibited consistent moisture levels of approximately 5% and water activity (a_w_) of 0.3. Furthermore, the viable cell counts after encapsulation remained above 90% for all three strains evaluated. All these results are summarized in Table 1.

### 2.2. Morphology of Microcapsules

The shape of the microcapsules obtained was observed by scanning electron microscopy. Since the formulation of the three synbiotics was the same, the resulting microcapsules had a similar average size: 169.8 ± 95.8 μm for *Lacticaseibacillus rhamnosus* LM07, 176.1 ± 95.8 μm for *Lactiplantibacillus plantarum* LM 19, and 164.9 ± 92.4 μm for *Levilactobacillus brevis* LBH1070.

**Figure 1:**
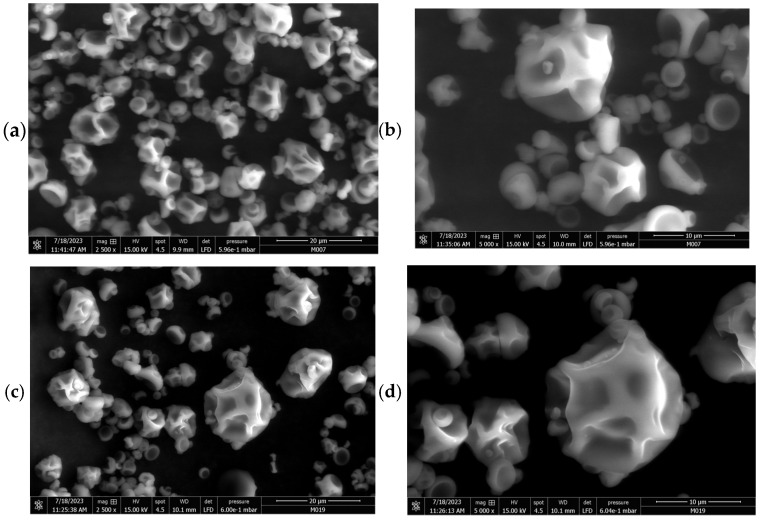

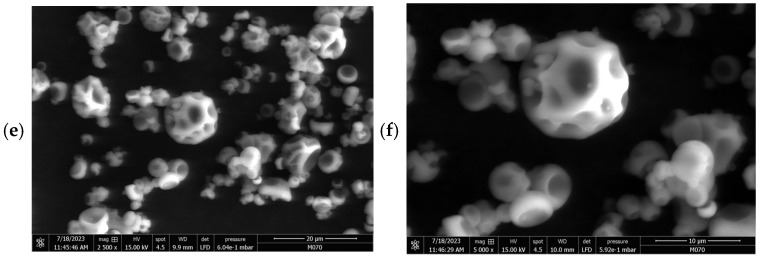
Scanning electron microscopy of microcapsules obtained from synbiotic formulations at 2500× (**left**) and 5000× (**right**). (**a**,**b**): *Lacticaseibacillus rhamnosus* LM07. (**c**,**d**): *Lactiplantibacillus plantarum* LM 19. (**e**,**f**): *Levilactobacillus brevis* LBH1070.

Micrographs showed that the microcapsules had a surface with no visible cracks or fractures but with dents (Figure 1).

### 2.3. Cell Viability During Storage

**Figure 2:**
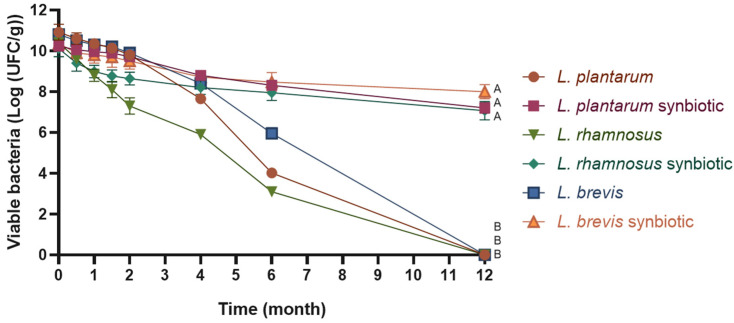
Free *Lactobacillus* and synbiotic viability during storage (4 °C for free strains and room temperature for synbiotics). The graph was plotted as mean ± SD, *n* = 3. Different letters indicate significant differences (*p* ≤ 0.05) between free and microencapsulated *Lactobacillus* after 12 months of storage at room temperature.

The cell viability of *L. rhamnosus*, *L. plantarum*, and *L. brevis* over 12 months of storage in free (4 °C) and microencapsulated forms (25 °C) is shown in Figure 2. The free strains (non-microencapsulated) exhibited a decline in viability to below 10^7^ CFU after the fourth month. By the 12th month, viability was lost entirely for all three free strains at 4 °C. In contrast, the microencapsulated strains (synbiotics) stored at room temperature experienced a reduction of only three logarithmic cycles, maintaining levels of 10^8^ for *L. brevis* (70.38% of initial viability) and 10^7^ for *L. rhamnosus* (69.93%) and *L. plantarum* (77.22%) after 12 months. These results demonstrate that microencapsulation significantly contributed to preserving cell viability over time.

### 2.4. Probiotic Properties

Subsequently, some probiotic properties of the synbiotics of *L. rhamnosus*, *L. plantarum*, and *L. brevis* were evaluated, such as their adhesion property (by evaluating their hydrophobicity and their auto-aggregation), their tolerance to digestion conditions (where phenol resistance, pH, bile salts, and in vitro digestion were evaluated), and their antioxidant capacity (by evaluating their DPPH and ABTS radical scavenging activity), to determine the effect of microencapsulation on the properties of each strain.

#### 2.4.1. Adhesion Properties: Auto-Aggregation and Hydrophobicity

**Figure 3:**
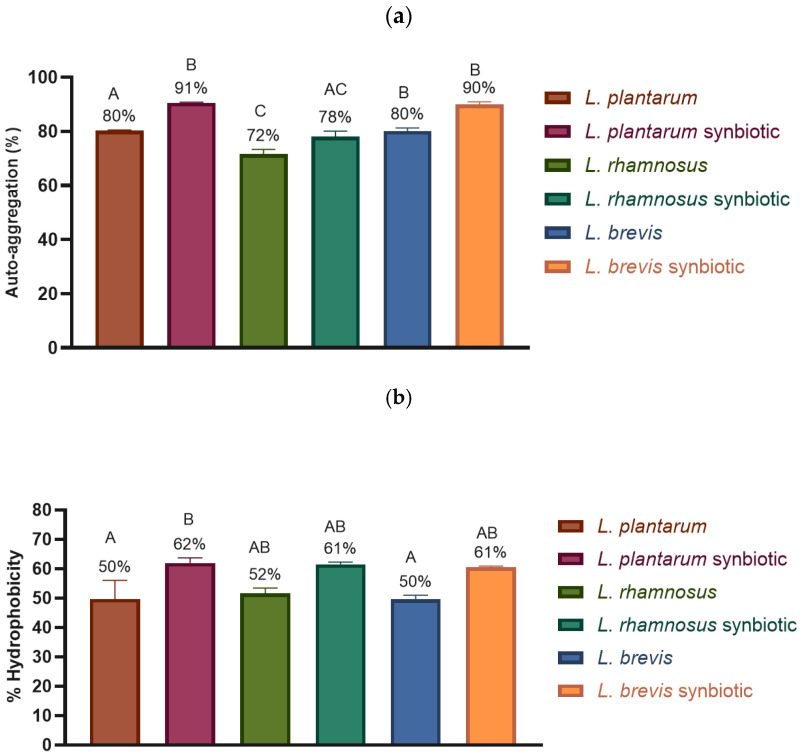
Effect of synbiotic microencapsulation of *Lacticaseibacillus rhamnosus* LM07, *Lactiplantibacillus plantarum* LM 19, and *Levilactobacillus brevis* LBH1070 on their adhesion properties. (**a**) Auto-aggregation. (**b**) Hydrophobicity. The graphs were plotted as mean ± SD, *n* = 3. Different letters indicate significant differences (*p* ≤ 0.05).

Figure 3 shows the auto-aggregation and hydrophobicity properties of the *Lactobacillus*. The microencapsulated strains showed higher adhesion properties compared to the free strains in both auto-aggregation and hydrophobicity. Although an increase was observed in all three strains, according to the statistical analysis, only *L. plantarum* showed a statistically significant difference.

#### 2.4.2. Tolerance to Digestion Conditions

Probiotics must withstand gastrointestinal conditions to survive in sufficient quantities and colonize the body. If these properties are compromised, their beneficial effects may be reduced. To evaluate the ability of our *Lactobacillus* strains to survive digestion, we assessed their growth under various conditions: exposure to phenol (a bacteriostatic byproduct of aromatic amino acid deamination), different pH levels (ranging from pH 1, representing the stomach, to pH 8, corresponding to the ileum), exposure to bile salts (involved in fat digestion and absorption, which may inhibit Gram-positive bacteria), and a simulated digestion process where *Lactobacillus* strains were exposed to general digestive conditions such as pH, temperature, and enzymatic activity.

- Phenol

**Figure 4:**
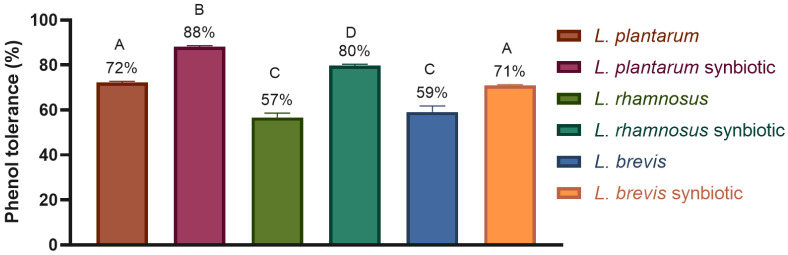
Effect of synbiotic microencapsulation of *Lacticaseibacillus rhamnosus* LM07, *Lactiplantibacillus plantarum* LM 19, and *Levilactobacillus brevis* LBH1070 on their tolerance to phenol, 0.04%. The graph was plotted as mean ± SD, *n* = 3. Different letters indicate significant differences (*p* ≤ 0.05).

Figure 4 shows the phenol tolerances of *Lactobacillus.* The growth of the strains in free form was 57–72%, while the resistance to phenol of the microencapsulated strains was significantly better, obtaining a value of 71–88%. This could indicate that the microencapsulation of the strains could help prevent the bacteriostatic effect of phenol.

- pH

**Figure 5:**
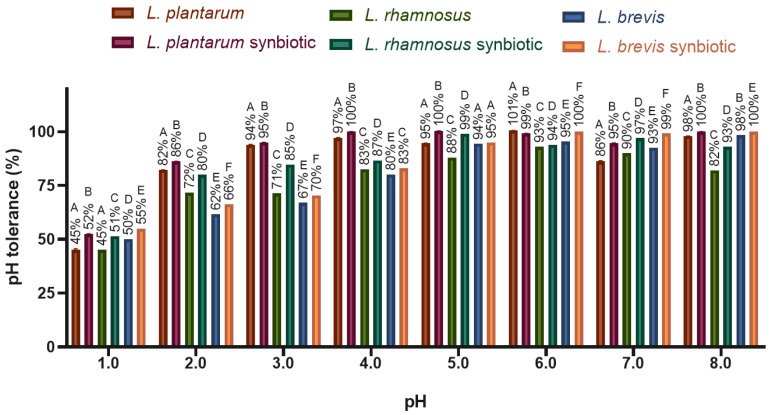
Free *Lactobacillus* and synbiotic tolerance to different pH levels. The graph was plotted as mean ± SD, *n* = 3. Different letters indicate significant differences (*p* ≤ 0.05) between every *Lactobacillus* strain growing in each pH value.

All three *Lactobacillus* strains showed a decrease in pH tolerance proportional to the pH value (Figure 5). However, even at the lowest pH (pH 1), bacterial growth remained above 40% in all cases. At the other pH values, growth remained above 60%, indicating that the *Lactobacillus* strains are tolerant to the different pH levels they may encounter during digestion. Synbiotics exhibited a higher growth percentage compared to free bacteria.

- Bile salt tolerance

**Figure 6:**
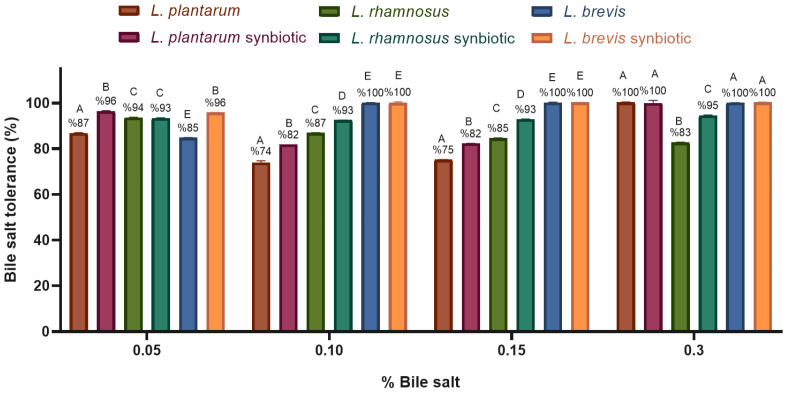
Free *Lactobacillus* and synbiotic tolerance to different bile salt concentrations. The graph was plotted as mean ± SD, *n* = 3. Different letters indicate significant differences (*p* ≤ 0.05) between each *Lactobacillus* strain growing in each bile salt concentration.

Tolerance to bile salts was high in most cases and higher in microencapsulated strains compared to free strains. The concentration of bile salts does not seem to influence the growth of free or microencapsulated strains since the growth of all strains above 70% was observed in the four concentrations of bile salts evaluated (Figure 6).

- In vitro digestion

**Figure 7:**
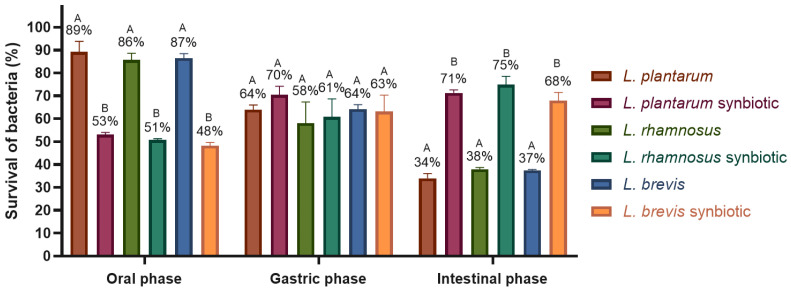
Free *Lactobacillus* and synbiotic viability after in vitro digestion. The graph was plotted as mean ± SD, *n* = 3. Different letters indicate significant differences (*p* ≤ 0.05) between each *Lactobacillus* strain after each in vitro digestion phase.

During the in vitro digestion process of the strains, it was observed that in the oral phase, the three free strains maintained a viability of 86–89% (Figure 7), while the synbiotics showed a 48–53% viability. These lower values in the oral phase were attributed to the fact that the microcapsules had not yet released the whole number of microencapsulated bacteria. This was confirmed in the gastric and intestinal phases, where the percentage of viable bacteria increased to 61–70% in the gastric phase and 68–75% in the intestinal phase for the synbiotics. In contrast, the free strains exhibited a decrease in viability during the intestinal phase, dropping to 34–38%. At the end of the entire digestion process, a viability of 7.28 ± 0.14 CFU/g was obtained for the *L. plantarum* synbiotic, 7.31 ± 0.36 CFU/g for the *L. rhamnosus* synbiotic, and 6.83 ± 0.30 CFU/g for the *L. brevis* synbiotic. In comparison, the free strains showed significantly lower viability, with 3.71 ± 0.23 CFU/mL for *L. plantarum*, 3.94 ± 0.07 CFU/mL for *L. rhamnosus*, and 4.04 ± 0.04 CFU/mL for *L. brevis*.

#### 2.4.3. Antioxidant Activity: ABTS and DPPH Free Radical Scavenging

**Figure 8:**
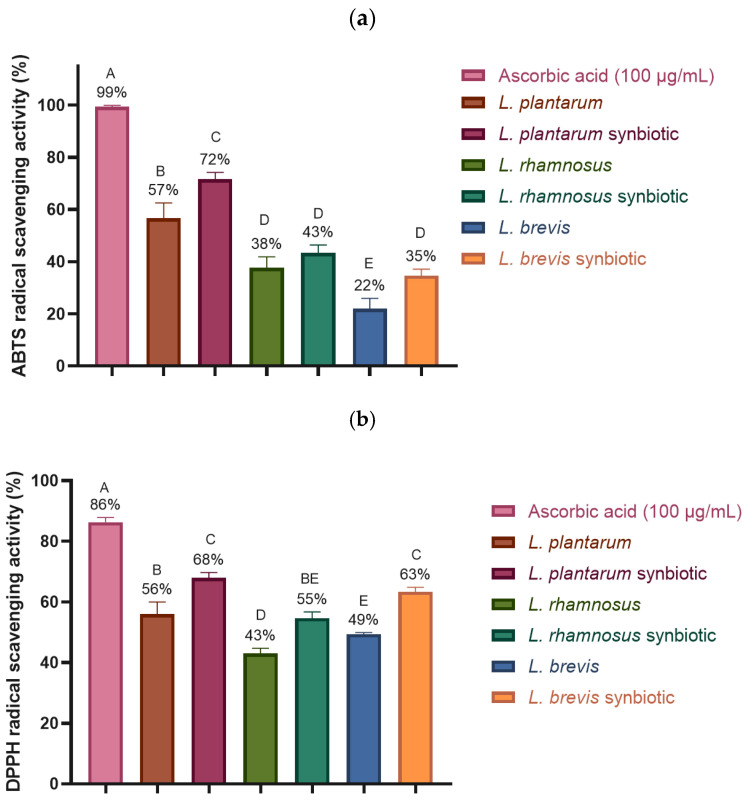
Effect of synbiotic microencapsulation of *Lacticaseibacillus rhamnosus* LM07, *Lactiplantibacillus plantarum* LM 19, and *Levilactobacillus brevis* LBH1070 on their antioxidant activity. (**a**) ABTS radical scavenging activity. (**b**) DPPH radical scavenging activity. The graphs were plotted as mean ± SD, *n* = 3. Different letters indicate significant differences (*p* ≤ 0.05).

The antioxidant activity of the strains was evaluated, and it was observed that the synbiotics showed a higher percentage of free radical inhibition for both ABTS and DPPH (Figure 8). In the case of the ABTS radical, the radical scavenging activity was 22–57% for the free strains, whereas the synbiotics showed a significant increase, reaching between 35% and 72% (Figure 8a). *L. plantarum* and its synbiotic were the strains with the highest percentages of radical inhibition, although this activity did not reach the 99% ABTS inhibition achieved by the ascorbic acid control. Regarding the DPPH radical, the free strains showed inhibition percentages ranging from 43% to 56%, while the synbiotics demonstrated an improved antioxidant capacity, with values ranging from 55% to 68% (Figure 8b). In this case, the synbiotics of *L. plantarum* and *L. brevis* showed the highest values, with no significant differences, after the control of ascorbic acid, which had an inhibition percentage of 86%.

## 3. Discussion

The yields obtained in the microencapsulation process were consistent with expectations for a spray-drying method and fell within the range reported by other authors. Avila-Reyes et al. [20] documented 65–74% microencapsulation efficiencies for *L. rhamnosus* in rice starch and 43–54% in inulin. Xavier dos Santos et al. [16] reported 81–98% yields for *Lactobacillus acidophilus* La-5 microencapsulated using inulin as the coating agent. On the other hand, Leylak et al. [23] reported a maximum yield of 48.36% for the optimization of *Lactobacillus acidophilus* La-5 encapsulation in mixtures of whey powder and gum arabic, attributing the low process efficiency to technical issues related to stickiness. Therefore, the results obtained for our three *Lactobacillus* synbiotics (~70%) suggest that the inlet and outlet temperature conditions, along with the use of a mixture of maltodextrin, gum arabic, and inulin, contributed to a low-tackiness product. This minimized powder retention in the drying chamber and in the outlet air pipe, facilitating the recovery of most solids.

According to Broeckx et al. [24] moisture levels around 4% are recommended to prevent deterioration reactions, such as lipid oxidation, and to ensure bacterial viability over extended periods. In addition, Huang et al. [25] mention that the optimal a_w_ range for probiotic storage is below 0.2. Similarly, Kalita et al. [26] indicate that an a_w_ value > 0.5 harms cell viability and may promote the growth of unwanted microorganisms. The values obtained in this study (5% moisture and a_w_ of 0.3) were slightly higher than the optimal values mentioned previously. This could be attributed to the presence of maltodextrin, as microcapsules containing maltodextrin, compared to other coating materials, can exhibit higher a_w_ values due to its smaller and more branched structure [26]. Although these values remain below the risk thresholds and indicate favorable conditions for microencapsulated products—supporting their stability and functionality during storage—future studies could explore adjustments to the coating materials to reduce these values further. Moayyedi et al. [27] mention that air temperature significantly affects the a_w_ of dried products. However, the temperature used in our spray-drying process was relatively high (70 °C), and further increases could compromise bacterial viability and reduce shelf life. This study did not thoroughly investigate the potential effects of higher drying temperatures on bacterial survival and long-term storage stability.

Huang et al. [25] reported that cell survival after the encapsulation process—commonly referred to as encapsulation efficiency—can be influenced during spray-drying by various factors, such as the residence time of microcapsules in the drying chamber, inlet and outlet temperatures, spray pressure, total solids content, and the properties of the coating materials. Due to variations in processing conditions and combinations of wall materials with probiotics, different efficiency percentages have been reported in the literature: 65–89.15% for *Lactobacillus acidophilus* NCDC 016 encapsulated in maltodextrin and gum arabic [10]; 62.6–86.5% for *Lactobacillus acidophilus* La-5 using inulin [16]; 99.46% for *Lb. acidophilus* CRL1014, 100% for *Lb. fermentum* CRL1446, and 96.19% for *Lb. johnsonii* CRL1231 with a combination of sodium alginate, inulin, and maltodextrin [15]; 93.95% for *Lactobacillus acidophilus* LA-5 using whey powder and gum arabic [23]; and 94.61% for *L. lactis* R7 with whey and inulin [19]. In our study, the high cellular viability observed after the process—with survival rates exceeding 90%—demonstrates that the short heat exposure times employed during spray-drying did not significantly affect bacterial viability. Additionally, the results emphasize that the encapsulation conditions, combined with the careful selection and proportions of wall materials, provided adequate protection and stability to the bacteria. These findings confirm the method’s reliability, validating its ability to preserve probiotic strains under controlled conditions and suggesting that it could be a viable strategy for developing more stable and effective probiotic products.

Synbiotic micrographs revealed the presence of dents in the microcapsules, a common feature of the spray-drying process caused by the contraction of particles due to temperature fluctuations during the procedure [20,26,27]. Kuck and Noreña [28] attribute this phenomenon in gum arabic microparticles to the protein fraction of the molecular structure. They suggest that this structure is closely related to encapsulation efficiency, as the small protein chain, covalently linked to the carbohydrate chain, acts as an excellent film-forming agent, resulting in effective encapsulation. Moreover, the absence of free *Lactobacillus* on the surface of the particles in all cases indicates that the bacteria were successfully encapsulated within the microparticles [10,29].

The evaluation of cell viability during storage further validates the efficacy of the microencapsulation process. The three strains (*L. rhamnosus*, *L. plantarum*, and *L. brevis*) stored at room temperature lost only three logarithmic units over 12 months, maintaining 69.93–77.22% of their initial viability. In contrast, the free strains exhibited complete loss of viability during the same period. This suggests that the encapsulation process, which produced powder products with low moisture content and low water activity (a_w_), prevented cell death during storage and extended the viability of the strains. Moreover, the viability of the microencapsulated strains remained above 10^7^ CFU/g, meeting the minimum concentration generally accepted for probiotics to provide health benefits [30,31]. These results demonstrated a good survival rate after 12 months, especially compared to previous studies reporting lower survival rates over shorter storage durations. For instance, *L. fermentum* CRL1446 and *L. johnsonii* CRL1231 were preserved above 10^6^ CFU/g over 12 months at 4 °C, while *L. acidophilus* CRL1014 maintained this level for only 4 months [15]. Similarly, *L. acidophilus* NCIM 2660 retained 72.4% of its viability after three weeks at room temperature; *L. bulgaricus* NCIM 2056: 87.0%; *L. fermentum* NCIM 2156: 74.5%; and *L. plantarum* NCIM 2083: 84.6% [32]. Additionally, *L. plantarum* BM-1 maintained 68.63% viability after two months of storage at room temperature [17], while *Lactococcus lactis* subsp. *lactis* R7 remained above log 8 CFU/g after six months at room temperature [19]. Even though our results are promising, additional studies under varying environmental conditions are necessary to better understand the stability and functionality of microencapsulated probiotics in real-world conditions.

After microencapsulation, an increase in the auto-aggregation and hydrophobicity properties of the *Lactobacillus* strains was observed. According to Sánchez and Tromps [33], bacterial hydrophobicity is classified as low (0–29%), medium (30–50%), and high (51–100%). Following this classification, the microencapsulation process increased the hydrophobicity of the strains from medium to high for *L. plantarum* and *L. brevis*, while *L. rhamnosus* maintained a high level in both cases, with an even higher value as a synbiotic. These results suggest that microencapsulation, in addition to protecting bacteria from unfavorable conditions, may contribute to enhancing the hydrophobicity and auto-aggregation properties of these bacterial strains. These adhesion properties are crucial for bacterial growth and colonization, as greater hydrophobicity and auto-aggregation increase the likelihood of bacteria interacting with the intestinal epithelial cells [33].

Regarding phenol tolerance, the results indicate that microencapsulation significantly enhances bacterial protection against this bacteriostatic compound. The greater tolerance observed in microencapsulated strains highlights the protective role of the encapsulating matrix, which likely acts as a physical barrier. This barrier reduces direct interaction between the bacteria and phenol, thereby improving survival.

Additionally, the three evaluated free and microencapsulated strains exhibited good resistance to various pH levels, including low pH. Although survival rates decreased under acidic conditions, they remained above 40%. This suggests that even the free cells showed good tolerance, which is one of the characteristics probiotics should have. Torres-Maravilla et al. [22] reported the survival of lactobacilli strains ranging from 41% to 89.93% after 180 min at pH 2. Similarly, Reale et al. [34] found that *L. rhamnosus*, *L. casei*, and *L. paracasei* strains were capable of surviving at pH 1.5 and 2.5 for 2 h. Moumita et al. [32] evaluated *Lactobacillus acidophilus* NCIM 2660, *Lactobacillus bulgaricus* NCIM 2056, and *Lactobacillus fermentum* NCIM 2165, reporting survival rates of 24%, 38%, and 34%, respectively, after 2 h of gastric exposure at pH 2. Some other authors had reported good resistance of free strains to low pH with an increase in the survival rate with microencapsulated strains. Xavier dos Santos et al. [16] reported survival rates of 23.55% and 77% for free and microencapsulated *Lactobacillus acidophilus* La-5 after exposure to gastric conditions at pH 2. Zhu et al. [17] observed that after 120 min in simulated gastric conditions, the cell count of free *L. plantarum* BM-1 decreased by approximately 1.4 log CFU/mL (~83% of the initial count), while the microencapsulated strain did not show any significant reduction. Arepally et al. [10] reported survival rates of 15% and 62% after 3 h at pH 1, 19% and 68% after 3 h at pH 1.5, and 21% and 71% after 3 h at pH 2 for free and microencapsulated *Lactobacillus acidophilus* NCDC 016, respectively. However, all these studies assessed bacterial survival under simulated gastric conditions, using acidic solutions to mimic the stomach environment. In contrast, our study evaluated strain growth in MRS broth, which provides the necessary nutrients for bacterial proliferation. However, given this limitation, we also conducted an in vitro digestion assay, which is discussed later in this article.

Similarly, bile resistance exceeded 70% for all strains, with microencapsulated strains exhibiting slightly higher resistance without significant differences. These findings align with previously reported properties of probiotic strains that were also microencapsulated. For instance, Zhu et al. [17] evaluated the tolerance of microencapsulated *L. plantarum* BM-1 to bile salts at a concentration of 0.5%, observing a reduction of only 0.1 log CFU/mL. Other researchers have studied strain tolerance when exposed to acidic conditions and bile salts during simulated gastrointestinal processes. Under these conditions, they reported that pH and bile presence did not significantly affect the microencapsulated strains’ viability [18,19]. The notable improvement in tolerance to both acidic pH and bile salts underscores the potential of this technology for developing more efficient delivery systems. These systems promote the stability and functionality of probiotics in the digestive tract. The increased survival could enhance colonization efficiency and probiotic activity, providing more significant health benefits than non-microencapsulated strains.

Furthermore, in vitro digestion results reveal significant differences in survival rates between free strains (34–38%) and microencapsulated strains (68–75%). This suggests that microencapsulation can delay the release of *Lactobacillus*, allowing it to occur during the intestinal phase and thereby increasing the likelihood of colonization and beneficial activity. This delayed release mechanism, attributed to the protective structure of the microcapsules, likely prevents premature exposure to adverse conditions such as low pH and enzymatic activity in the stomach, which would otherwise reduce bacterial viability. The protective effect of encapsulation has also been observed in other studies, where survival during digestion processes was higher in microencapsulated strains than in free strains. For example, microencapsulated *L. acidophilus* CRL1014, *L. fermentum* CRL1446, and *L. johnsonii* CRL1231 showed survival rates of 65%, 88%, and 71%, respectively, after the intestinal phase, while only 25%, 76%, and 48% of their free forms survived, respectively [15]. Similarly, microencapsulated *L. acidophilus* La-5 exhibited a significant increase in survival after digestion compared to the free strain, rising from 10% to 76% [16]. In another study, microencapsulated *L. acidophilus* NCDC 016 survived to 64.14% by the end of the intestinal phase, whereas the free strain survived only to 12.26% [10]. The increased resistance of microencapsulated strains to acidic pH, bile salts, and phenol indicates that microencapsulation significantly improves the survival of probiotic strains under adverse gastrointestinal conditions that might otherwise inhibit their growth. This improvement is likely due to the protective matrix shielding the bacteria from hostile environments and stress. These findings suggest that this technology could be applied to develop delivery systems for probiotics that are more resilient to the harsh conditions of the gastrointestinal tract. Such systems could potentially enhance probiotic viability, promote their colonization, and improve their effectiveness.

The antioxidant activity of probiotics has been previously reported. For instance, *Lactobacillus plantarum* RYPR1 demonstrated a 78.53% elimination of the ABTS radical [35]. Similarly, *L. plantarum* ZJ316 showed 59.82% elimination of the DPPH radical and 61.28% elimination of the ABTS radical [36]. In contrast, *Lactobacillus reuteri* MG505 and *Lactobacillus rhamnosus* MG316 achieved 33.5% and 22.2% elimination of the DPPH radical, respectively, and 55.9% and 50.0% elimination of the ABTS radical, respectively [37]. However, microencapsulation’s effect on probiotics’ antioxidant activity has not been previously reported. Nevertheless, Mounir et al. [38] mention that synbiotics can generate antioxidant compounds with more significant activity than probiotics alone, due to additive or synergistic effects. This is because prebiotics can improve the viability of probiotics, and in some cases, prebiotics themselves possess antioxidant properties or are used as substrates for probiotics to produce more antioxidant compounds [38]. The results obtained for ABTS and DPPH in this study suggest that microencapsulation may positively influence the antioxidant activity of the three *Lactobacillus* strains. This improvement in free radical scavenging ability could be related to the structure of the microcapsules, which not only protect the bacteria from unfavorable conditions but may enhance antioxidant activity, either in a complementary or in a synergistic way, due to the presence of hydroxyl groups in GA, MD, and I that are capable of donating electrons to reduce the radicals to a more stable form or to react with the free radicals to terminate the radical chain reaction [38]. Thus, synbiotic microencapsulation has the potential to enhance these strains’ health benefits by increasing their antioxidant activity. Considering this, future research could explore the mechanisms underlying the microencapsulation process and its interaction with bacterial strains to enhance antioxidant activity. Furthermore, to better understand the potential of microencapsulated probiotics in functional food development, future studies should prioritize in vivo models to evaluate their colonization efficiency and health benefits, particularly in improving gut health. These efforts could provide deeper insights into the practical applications of microencapsulation as an innovative technology for developing functional foods with health-promoting effects.

## 4. Materials and Methods

### 4.1. Potential Probiotic Strains and Growth Conditions

In the present research, three potential probiotic strains were used: *Lacticaseibacillus rhamnosus* LM07 and *Lactiplantibacillus plantarum* LM 19 from mezcal production from Oaxaca, México [21], and *Levilactobacillus brevis* LBH1070 from pulque from Tlaxcala, Mexico [22]. Strains were separately grown at 10% (*v*/*v*) in MRS broth (Man, Rogosa, and Sharpe, pH 6.5 Merck^®^, Darmstadt, Germany) and incubated for 24 h at 37 °C. The bacterial growth was centrifuged at 12,560× *g* for 7 min at 4 °C (Metrix^®^ Dynamica centrifuge, Velocity 18R, Sunderland, UK), washed twice with PBS (pH 6.9), and resuspended in PBS to obtain a concentration of 10^10^ colony-forming units (CFU)/mL.

### 4.2. Spray-Drying Microencapsulation

The formulation for the microencapsulation process was a combination of maltodextrin (5% MD) with gum arabic (10% GA) as wall materials and inulin (5% I) as a prebiotic to obtain a synbiotic effect. This formulation was determined through prior unpublished experimentation, detailed in European Patent Application No. EP24306864.0. The MD-GA-I solutions were inoculated, separately, with each *Lactobacillus* strain to obtain a final concentration of 10^11^ CFU/mL. These solutions were sprayed into a pilot-scale spray-dryer (Mobile Minor 2000, GEA) using a peristaltic pump (Watson-Marlow 520S) with a feed flow of 7–12 mL/min. The inlet temperature was 160 ± 1 °C and the outlet air temperature was 70 ± 2 °C, with a pressure of 2 bar [39]. After spray-drying, the encapsulation process yield, viability, bacterial survival, moisture content, and water activity were measured. The stability of the microcapsules was assessed by evaluating bacterial viability during storage. The survival rate on the day of spray-drying was compared to viability after 0.5, 1, 1.5, 2, 4, 6, and 12 months. Synbiotic samples were stored in glass containers inside a desiccator maintained at a constant relative humidity of 16% using silica gel (Hycel, Mexico). The desiccator was kept at room temperature (25 ± 2 °C). During storage, temperature and relative humidity (RH) were monitored with an Air Quality Monitor (INKBIRD, AQItech AK3, Shenzhen, China). Free strains were stored as liquid culture at refrigeration (4 °C).

The encapsulation process yield was calculated according to Equation (1) [16]:

(1) $$Yield\;(\%)=\frac{S}{S_{0}}\times 100$$

where S is the total of solids obtained after the spray-drying process (g), and S_0_ is the total of solids in the feed solution (before the spray-drying process) (g).

Bacterial survival after the spray-drying process was also calculated according to Equation (2) [16]:

(2) $$Bacterial\;survival\;(\%)=\frac{N}{N_{0}}\times 100$$

where N is the number of viable bacteria from microcapsules (log CFU/g), and N_0_ is the number of viable bacteria in the feed solution (before the spray-drying process) (log CFU/g).

Moisture content was determined using 0.5 g of sample in a moisture analyzer (Ohaus MB45, Parsippany, NJ, USA) for 10 min at 100 °C. Determinations were performed in duplicate [39].

Water activity was measured using a water activity analyzer (Graigar HBD5-MS2100Wa, Shenzhen, China). Determinations were performed in duplicate [39].

Bacterial viability in synbiotics was determined by diluting 0.5 g of synbiotic in 4.5 mL of PBS (pH 6.9) and vortexing the mixture until complete rupture of the microcapsules. For free strains, bacterial viability was determined directly using 1 mL of the bacterial solution [39].

### 4.3. Scanning Electron Microscopy (SEM)

Microcapsules were observed using a scanning electron microscope (FEI-ThermoFisher Scientific, ESEM Quanta FEG 250, Waltham, MA, USA). The analysis was performed under a voltage of 15 kV. The mean size of the microcapsules was calculated using ImageJ software (v. 1.52a, USA) by recording the length of 100 particles in the micrographs obtained with 2500× and 5000× magnification for each strain [39].

### 4.4. Adhesion Capacity

#### 4.4.1. Hydrophobicity

According to García-Hernández et al. [40], a bacterial suspension for each *Lactobacillus* strain was obtained and adjusted to an optical density = 1 (600 nm); this value was considered as the initial reading (A_0_). A total of 5 mL of this suspension and 1 mL of toluene were mixed in vortex for 2 min and poured into a separation funnel. After 1 h incubation at 37 °C, the absorbance (A) of the aqueous phase was determined in triplicate for each sample. Hydrophobicity (%H) was calculated according to Equation (3):

(3) $$Hydrophobicity\;(\%)=\frac{A_{0}-A}{A_{0}}\times 100$$

#### 4.4.2. Auto-Aggregation

According to Angmo et al. [41], a bacterial suspension for each *Lactobacillus* strain was obtained and adjusted to an optical density of 0.25 ± 0.05 (600 nm); this value was considered as the initial reading (A_0_). A total of 5 mL of this suspension was stirred in vortex for 10 s. After 24 h of incubation at 37 °C, absorbance was determined (A) in triplicate for each sample. Auto-aggregation was obtained according to Equation (4):

(4) $$Auto\text{-}aggregation\;(\%)=\frac{A_{0}-A}{A_{0}}\times 100$$

### 4.5. Tolerance to Digestion Conditions

#### 4.5.1. Phenol Resistance

According to Vizoso Pinto et al. [42], *Lactobacillus* strains were grown in MRS broth with and without 0.4% phenol (*v*/*v*). The determinations were carried out in triplicate. After 24 h of incubation at 37 °C, viable cells were counted, and the tolerance to phenol was calculated based on Equation (5):

(5) $$Phenol\;tolerance\;(\%)=\frac{{CFU/mL}_{MRS\;with\;phenol}}{{CFU/mL}_{MRS\;without\;phenol}}\times 100$$

#### 4.5.2. pH Tolerance

According to Castillo Arroyo et al. [43], *Lactobacillus* strains were grown in MRS broth at different pH values ranging from 1 to 8. The pH was adjusted using 1 N HCl and 1 N NaOH. The determinations were carried out in triplicate. After 24 h incubation at 37 °C, growth was measured by determination of optical density at 600 nm, and tolerance to pH was determined by Equation (6):

(6) $$pH\;tolerance\;(\%)=\frac{{OD}_{MRS\;with\;X\;pH}}{{OD}_{MRS\;with\;normal\;pH}}\times 100$$

#### 4.5.3. Bile Salt Tolerance

According to Castillo Arroyo et al. [43], *Lactobacillus* strains were grown in MRS broth with four salt concentrations (0.05, 0.1, 0.15, and 0.3% *w*/*v*). The determinations were carried out in triplicate. After 24 h incubation at 37 °C, viable cells were counted, and bile salt tolerance was determined by Equation (7):

(7) $$Bile\;salts\;tolerance\;(\%)=\frac{{CFU/mL}_{MRS\;with\;x\;bile\;salts\;concentration}}{{CFU/mL}_{MRS\;without\;bile\;salt}}\times 100$$

#### 4.5.4. In Vitro Digestion

In vitro digestion was simulated by preparing simulated salivary fluid (SSF), simulated gastric fluid (SGF), and simulated intestinal fluid (SIF) as was described in Minekus et al. [44]; lysozyme, pepsin, pancreatin, and bile salts were added at the concentration described in Vizoso Pinto et al. [42]; the experiment was carried out in duplicate for each sample, and, after each phase, 100 µL aliquots were taken to quantify viable bacteria. To simulate the oral phase, a final ratio of sample to SSF of 1:9 was targeted according to the proportions of microencapsulated and free cells used by Russo et al. [15]. A total of 0.5 mL of the *Lactobacillus* strain or 0.5 g of the synbiotic was added to 4.5 mL of SSF with lysozyme (0.1 g/L, with an activity of 10,728 U/mL in the final mixture) and incubated for 2 min at 37 °C and 80 rpm. Then, to simulate the gastric phase, the mixture obtained was added to 7.5 mL of SGF with pepsin (3 g/L, with an activity of 9000 U/mL in the final mixture) and incubated for 2 h at 37 °C and 80 rpm. Finally, to simulate the intestinal phase, 5 mL of gastric phase was mixed with 40 mL of SIF with pancreatin (1 g/L, with a trypsin activity of 200 U/mL in the final mixture) and bile salts (5 g/L) and incubated for 2 h at 37 °C and 80 rpm. The survival rate (% S) compared to the initial count before in vitro digestion was calculated according to Equation (8) [16]:

(8) $$Survival\;rate\;(\%)=\frac{N}{N_{0}}\times 100$$

where N is the number of viable cells (Log CFU/mL) after each phase (oral, gastric, or intestinal) and N_0_ is the initial number of viable cells before in vitro digestion (Log CFU/mL).

### 4.6. Antioxidant Activity

#### 4.6.1. ABTS Free Radical Scavenging

According to Kim et al. [37], a stock solution of the ABTS^+^ cationic radical was prepared via the mixture of 25 mL of ABTS 7 mM with 25 mL of potassium persulfate 2.45 mM at room temperature for 24 h. Afterward, the stock solution was diluted with distilled water up to an absorbance value of 0.70 ± 0.05 measured at 734 nm. Then, 500 μL of each sample (free strain adjusted to an optical density = 1 (600 nm) or 0.1 g of synbiotic diluted in 1 mL PBS) was added to 1000 μL of the ABTS^+^ solution and incubated for 10 min in the dark at room temperature. Ascorbic acid (100 μg/mL) was used as a positive control. The determinations were carried out in triplicate. Antioxidant capacity was measured at 734 nm and calculated with Equation (9):

(9) $$ABTS\;inhibition\;(\%)=\frac{A_{0}-A_{m}}{A_{0}}\times 100$$

where A_0_ is the initial absorbance and A_m_ is the absorbance of the sample.

#### 4.6.2. DPPH Free Radical Scavenging

According to Wu et al. [36], 1 mL of a DPPH solution (0.2 mM) was added to 1 mL of the sample (free strain adjusted to an optical density = 1 (600 nm) or 0.1 g of synbiotic diluted in 1 mL PBS). Absorbance was read at 517 nm after 30 min incubation in the dark at room temperature. Ascorbic acid (100 μg/mL) was used as a positive control. The determinations were carried out in triplicate. Antioxidant capacity was calculated using Equation (10):

(10) $$DPPH\;inhibition\;(\%)=\frac{A_{0}-A_{m}}{A_{0}}\times 100$$

where A_0_ is the initial absorbance and A_m_ is the absorbance of the sample.

### 4.7. Statistical Analysis

The results are presented as the mean ± standard deviation. GraphPad Prism 8 software (GraphPad Software Inc., Boston, MA, USA) was used to perform the one-way analysis of variance (ANOVA) with a significance level of 0.05. The Tukey test was used to compare the means.

## 5. Conclusions

The strains previously isolated from fermented Mexican beverages showed promising probiotic properties. Furthermore, the microencapsulation of these strains to form their respective synbiotics appears to enhance their antioxidant activity and improve their tolerance to adverse conditions, especially those during the in vitro digestion process. This microencapsulation technique boosts the survival rate of the strains compared to the non-microencapsulated, ensuring that a higher proportion of viable bacteria reach the colonization site. These findings underscore the potential of these strains in the development of functional foods, offering new opportunities to provide additional health benefits through the incorporation of probiotics and synbiotics into food products.

## 6. Patents

The authors have submitted a patent application for the microencapsulation process described in this study (European Patent Application No. EP24306864.0), which is currently under review.

## Figures and Tables

**Table 1:** Characteristics of synbiotics obtained by spray-drying microencapsulation.

Strain		*L. rhamnosus* LM07	*L. plantarum* LM19	*L. brevis* LBH1070
Moisture content (%)		5.1 ± 0.9	5.0 ± 0.6	5.4 ± 0.7
Water activity (a_w_)		0.324 ± 0.115	0.312 ± 0.130	0.317 ± 0.122
Survival of bacteria	Log (CFU/g)	10.11 ± 0.40	10.23 ± 0.24	10.36 ± 0.36
	(%)	91.9 ± 3.6	93.0 ± 2.8	94.2 ± 4.1

Results are expressed as the mean ± standard deviation, *n* = 4.

## Data Availability

The data presented in this study are available upon reasonable request from the corresponding author, as they are subject to an ongoing patent application.

## Abbreviations

The following abbreviations are used in this manuscript:

CFU	Colony-forming unit
Aw	Water activity
MRS	Man, Rogosa, and Sharpe medium
PBS	Phosphate buffered saline
GA	Gum arabic
MD	Maltodextrin
I	Inulin
SSF	Simulated salivary fluid
SGF	Simulated gastric fluid
SIF	Simulated intestinal fluid
